# Supplementary material for: A putative structural mechanism underlying the antithetic effect of homologous RND1 and RhoD GTPases in mammalian plexin regulation
Source: eLife. 2021 Jun 11;10:e64304. doi: 10.7554/eLife.64304 (PMC8219378; doi:10.7554/eLife.64304)
Supplement: Supplementary file 1. [file elife-64304-supp1.docx]

**(Supplementary File 1)**

**Supplementary File 1. Diffraction data and structure refinement statistics.**

| **Data collection** |  |
| --- | --- |
| Space group | P3_1_ |
| Cell dimensions |  |
| a, b, c (Å) | 82.8, 82.8, 136.8 |
| α, β, γ (°) | 90, 90, 120 |
| Resolution (Å) | 50.0-3.10(3.15-3.10) |
| R_sym_ (%) | 7.5(51.4) |
| R_pim_(%) | 5.9(46.5) |
| *I*/σ | 23.2(1.3) |
| CC_1/2_^#^ | 0.843 |
| Completeness (%) | 99.2(91.2) |
| Redundancy | 5.4(3.5) |
| **Refinement** |  |
| Resolution (Å) | 39.63-3.10(3.26-3.10) |
| No. reflections | 18763 |
| *R*_work_/*R*_free_ (%) | 21.5(35.9)/26.6(37.6) |
| No. atoms |  |
| Protein | 4382 |
| Ligand/ion | 68 |
| Water | 8 |
| B-factors |  |
| Protein | 123.6 |
| Ligand/ion | 115.9 |
| Water | 85.1 |
| R.m.s deviations |  |
| Bond lengths (Å) | 0.002 |
| Bond angles (°) | 0.49 |
| Ramanchandran plot |  |
| Favored (%) | 97.1 |
| Allowed (%) | 2.9 |
| Disallowed (%) | 0 |
| Rotamer outlier(%) | 0 |
| All-atom clashscore | 5.88 |

*Numbers in parenthesis are for the highest resolution shell. # CC_1/2_ values shown are for the highest resolution shell.
